# Reversible inhibition of the basal ganglia prolongs repetitive vocalization but only weakly affects sequencing at branch points in songbirds

**DOI:** 10.1093/texcom/tgad016

**Published:** 2023-08-17

**Authors:** Hisataka Fujimoto, Taku Hasegawa

**Affiliations:** Department of Anatomy, Kawasaki Medical School, 577 Matsushima, Kurashiki, Okayama 701-0192, Japan; Laboratory for Imagination and Executive functions, RIKEN Center for Brain Science, 2-1 Hirosawa, Wakoshi, Saitama 351-0198, Japan

**Keywords:** Area X, lateral magnocellular nucleus of the anterior neostriatum, repetition, songbird

## Abstract

Although vocal signals, including languages and songbird syllables, are composed of a finite number of acoustic elements, diverse vocal sequences are composed of a combination of these elements, which are linked together by syntactic rules. However, the neural basis of syntactic vocalization generation remains poorly understood. Here, we report that inhibition using tetrodotoxin (TTX) and manipulations of gamma-aminobutyric acid (GABA) receptors within the basal ganglia Area X or lateral magnocellular nucleus of the anterior neostriatum (LMAN) alter and prolong repetitive vocalization in Bengalese finches (*Lonchura striata var. domestica*). These results suggest that repetitive vocalizations are modulated by the basal ganglia and not solely by higher motor cortical neurons. These data highlight the importance of neural circuits, including the basal ganglia, in the production of stereotyped repetitive vocalizations and demonstrate that dynamic disturbances within the basal ganglia circuitry can differentially affect the repetitive temporal features of songs.

## Introduction

The vocalization of complex and stereotyped phonemes requires precise neural control, which is mediated by the modulation of excitatory and inhibitory activities in higher-order neural circuits. Learned and stereotyped vocalizations for social communication are utilized by songbirds, including Bengalese finches (*Lonchura striata var. domestica*). Such vocalizations are regulated by activity in higher cortical nuclei. HVC (used as a proper name; Nottebohm et al. 1976; Aronov et al. 2008) is analogous to the supplementary motor cortex in mammals, whereas Area X is homologous to the mammalian basal ganglia and sends information to the lateral magnocellular nucleus of the anterior neostriatum (LMAN) via a thalamic nucleus (Reiner et al. 2004; Brainard and Doupe 2013; Pfenning et al. 2014). The information processed in the HVC is transmitted by two segregated types of projection neurons (Dutar et al. 1998; Mooney 2000): HVC_RA_ neurons that project to the robust nucleus of the arcopallium (RA), which relay information to the motor neurons for vocal organs (Nottebohm et al. 1976), and HVC_X_ neurons, which innervate Area X of the basal ganglia (Nottebohm et al. 1982) and are involved in vocal plasticity (Scharff and Nottebohm 1991; Brainard and Doupe 2000). HVC_X_ neurons encode syntactical motor signals for song production (Fujimoto et al. 2011). A previous study reported that the deletion of HVC_X_ did not alter the syntactic song structure, especially in the crystalized song of adult zebra finches (Sánchez-Valpuesta et al. 2019). However, the involvement of the songbird basal ganglia in the temporal regulation of syntactic vocalization, including repetitive syllables, remains unclear.

To date, most studies on the function of the song system (involving HVC, RA, Area X, and LMAN; Fujimoto et al. 2015) have been conducted in zebra finches (*Taeniopygia guttata*). However, adult zebra finches produce songs in a highly stereotypical manner; that is, syllable sequences have little or no variability across song renditions (Funabiki and Konishi 2003). By contrast, Bengalese finches (*Lonchura striata var. domestica*) produce variable syllable sequencing across renditions (Okanoya and Yamaguchi 1997; Okanoya 2004; Sakata and Brainard 2006, 2008; Fujimoto et al. 2011). Thus, a song possesses nodes at which the transitions vary (“branch points”) and a variable number of consecutively repeated syllables. Therefore, evaluation of how inhibition in the basal ganglia controls not only syllable structure and timing but also complex syllable sequencing is feasible in Bengalese finches. A previous study examined how context-dependent changes in syllable sequencing are linked to context-dependent changes in early growth response factor 1 (EGR-1) expression in basal ganglia circuits from Bengalese finches (Matheson et al. 2016). We evaluated the contribution of Area X and LMAN inhibition to song regulation by locally manipulating the inhibitory system in this study. We infused either tetrodotoxin (TTX), muscimol (a gamma aminobutyric acid subtype A [GABA_A_] receptor agonist), or gabazine (a GABA_A_ receptor antagonist), and assessed the changes in the temporal song syntax in adult Bengalese finches. Because inhibition of neural activity using TTX or muscimol, and facilitation using gabazine, can affect the song syntax in opposite manners, we examined how these drugs affect syllable repetition and syllable branch point.

## Materials and methods

### Experimental animals and data collection

Male Bengalese finches (*n* = 14; age range: 2.1–3.9 years) were raised in a breeding colony at the Kawasaki Medical School. All birds were maintained on a 14:10-h light-dark cycle and were provided with food and water ad libitum. All the procedures were approved by the Kawasaki Medical School Animal Care and Use Committee (approval number: 16-080), in accordance with the National Institutes of Health Guide for the Care and Use of Laboratory Animals for experiments. The birds were individually housed in a sound-attenuating chamber. Songs were recorded using microphones positioned above the songbird cages, and a computerized, sound-activated recording system was used to detect and digitize them (OCTA-CAPTURE, Roland, Japan). Recorded songs were digitally filtered at 0.5–8 kHz for analysis.

### Microdialysis probe implantation

Birds were initially anesthetized with an intramuscular injection of ketamine and xylazine. The birds were subsequently placed in a stereotaxic device, and microdialysis probes (CMA 7; Harvard Apparatus, Hollison, MA, USA) (Andalman and Fee 2009; Hamaguchi and Mooney 2012; Isola et al. 2020) were implanted bilaterally into the LMAN or Area X. The probes were fixed and secured in place using dental cement, and electrophysiological methods were used to identify the bursting activity that characterizes Area X or LMAN in anesthetized birds. The coordinates of Area X (4.5 mm rostral, 1.8 mm lateral) and LMAN (4.9 mm rostral, 1.85 mm lateral) were determined using the caudal edge of the bifurcation of the midsagittal sinus as a point of reference. After 5–7 days of recovery from the surgery, the reservoir system allowed for passive diffusion of TTX, muscimol, or gabazine in the freely behaving birds (Andalman and Fee 2009; Hamaguchi and Mooney 2012; Tanaka et al. 2016). Each day, the probes on both sides were slowly filled with 40 μL of 0.025 M phosphate-buffered saline (PBS, pH 7.4). The inlet and outlet tubes were outfitted with caps to minimize fluid evaporation. Because the insertion of the probes in and around Area X or LMAN could cause transient changes in the song, experimental infusions proceeded after the number of bouts had returned to the original state (the same mean ± standard deviation exhibited before surgery).

### Drug infusions

To assess the contribution of vocal motor control inhibition, birds were infused with TTX (2.5 or 5.0 μM; Sigma), muscimol (0.1, 0.2, or 1.0 mM; Sigma), or gabazine (0.05, 0.1, or 0.2 μM; Sigma). These concentrations have been used to manipulate neural activity in various regions of the songbird brain (Rosen and Mooney 2000; Stepanek and Doupe 2010; Warren et al. 2011; Hamaguchi and Mooney 2012; Tian and Brainard 2017; Isola et al. 2020). The drug doses were preliminarily explored through gradual daily increases. The maximum doses were determined as those that elicited several bouts that surpassed that of the preoperative state by greater than 10%. The statistical analysis was performed across sessions in which the same dose was used. Drug infusion sessions had a daily frequency, and each session was separated by at least one control session in which PBS was infused instead. Birds were infused within an hour (range: 20–60 min) before the lights-on phase of the light-dark cycle, and at the same time of the day, to control for the diurnal variation in the song syntax, including repetition (Scharff and Nottebohm 1991; Kobayashi et al. 2001; Isola et al. 2020). Solutions were washed with 0.025 M PBS 14 h following the infusion. Diurnal variations with drug infusions were examined in terms of the number of song bouts and the change in syllable repetition (Supplementary Fig. 1). Although the number of bouts tended to be lower during the 6 h after muscimol and gabazine infusions compared with PBS infusions, the number of bouts with TTX infusions were relatively stable. Additionally, diurnal variation in changes of repetition number was relatively small. Hence, birdsongs recorded during the entire day were combined for the analysis. After it was verified that drug infusion affected song vocalization, subsequent experimental infusions were separated by additional control infusion sessions with PBS (range: 1–3 days) until the number of song bouts returned to the normal state (the same mean ± standard deviation exhibited before surgery).

### Song analysis

Bengalese finch songs consist of acoustic elements that are arranged in specific sequences. For description and analysis, individual acoustic elements, or syllables, were separated from each other by at least 5 ms of silence (Okanoya and Yamaguchi 1997; Sakata and Brainard 2008; James and Sakata 2014). The labeling and analysis of the syllables were identical to those performed in previously published studies (Sakata and Brainard 2006; Sakata and Brainard 2008; Warren et al. 2012; James and Sakata 2014, 2015). All the syllables were initially identified using a linear support vector machine (Tachibana et al. 2014), and confirmed and corrected as needed. Briefly, following amplitude-based syllable segmentation, the syllables were manually labeled based on the visual inspection of spectrograms using custom-written MATLAB software (MathWorks Inc., Natick, MA, USA). Acoustic features (syllable duration, mean frequency, mean amplitude, and spectral and amplitude entropy) (Sakata and Brainard 2006; Wohlgemuth et al. 2010; Piristine et al. 2016; James and Sakata 2017) for each syllable in the song were analyzed and used to determine the syllable types using a linear support vector machine. Following automatic syllable identification, all the results were manually confirmed.

To quantify the experimental changes in syllable structure, the above-mentioned acoustic features of syllables after TTX, muscimol, or gabazine infusion were compared with those of the same syllables after PBS infusions. In addition, a coefficient of variation of fundamental frequency (CV of FF) was quantified for syllables with harmonic components with no or little frequency modulation (Kao et al. 2005; Isola et al. 2020). A harmonic component in each syllable was manually identified (range: 20%–80%), and the autocorrelation function of the sound waveform was computed. Fundamental frequency was defined as the reciprocal of the lag of the highest peak (excluding the peak at 0 ms lag) in the autocorrelation function, and the CV of FF was calculated across syllables of the same type for each experimental session.

To examine whether drug infusion regulates syllable sequencing, we analyzed two aspects of Bengalese finch song syntax: repetition number of the repetitive syllables, and syllable sequencing at branch points (Okanoya and Yamaguchi 1997; Jin 2009). A Bengalese finch song consists of syllables arranged in variable sequences known as branch points (Sakata and Brainard 2006, 2009; Warren et al. 2012) and repetitive syllables (Kobayashi et al. 2001; Fujimoto et al. 2011; Kubikova et al. 2014). In a branch point, a syllable is probabilistically followed by various other syllables. To compute transition entropy, we calculated the probability of different syllable transitions immediately following the branch point sequence, while syllable repetition was considered a single repeated syllable (Zhang et al. 2017). For each branch point, the sequence variability was quantified as the transition entropy:


$$ \mathrm{transition}\ \mathrm{entropy}=\Sigma -{\mathrm{p}}_{\mathrm{i}}\times \log\ \left({\mathrm{p}}_{\mathrm{i}}\right) $$

where the sum is the overall possible transitions, and p_i_ is the probability of the *i*th transition across all the songs (Gentner and Hulse 2000; Gil and Slater 2000; Sakata and Brainard 2006; Sakata and Brainard 2008; James and Sakata 2014, 2015). In each brain point, infrequent transitions (<5%) were excluded from the entropy calculation. Branch points with transitions that are closer to a uniform probability have higher transition entropy scores. The syllable repetition numbers in the sequences were counted as consecutive syllable repetitions.

### Statistical analyses

To analyze the transition entropy and CV of FF, the difference with the PBS infusion session results was used: average value under drug infusion − average value under PBS infusion. For the syllable repetition numbers and other acoustic features of syllables, the differences were normalized using the PBS infusion session results as reference: (average value under drug infusion − average value under PBS infusion)/average value under PBS infusion. The syllable repetition numbers in the sequences were analyzed for the syllable with the highest repetition number in each bird (except in the specific case of Supplementary Fig. 7).

Because syllable sequences and acoustic features were highly variable across individual birds and their syllables, the Kruskal–Wallis ANOVA test and Tukey HSD post-hoc test were used to assess the statistical significance of the effects of drugs on each syllable for syllable repetition numbers and each branch point for transition entropy (Tables 1 and 2 and Supplementary Fig. 5). Additionally, to examine the tendency of the effect of drugs across birds, the Kruskal–Wallis ANOVA test and Tukey HSD post-hoc test were conducted on syllable repetition numbers and transition entropy averaged across sessions (Figs. 2 and 4). A similar analysis was performed for the acoustic features of syllables; that is, the averages of durations, amplitude, entropy, and mean frequency, and CVs of FF were calculated in each experimental session with drug infusion and normalized with those recorded in control (PBS) sessions. Subsequently, the Kruskal–Wallis ANOVA test and Tukey HSD post-hoc test were used to assess the effects of drugs on each syllable (Supplementary Fig. 6).

**Table 1:** Demographics of the Bengalese finches using microdialysis probe implantations in bilateral LMAN.

Bird ID	Number of repeated syllable types	Number of branch points	Days recorded under infusion (Drug concentration)				Number of song bouts recorded under infusion				Number of syllables in repetitions under infusion Average ± SD				Number of branch points where transition entropy changes significantly^a^		
			PBS	TTX	Muscimol	gabazine	PBS	TTX	muscimol	gabazine	PBS	TTX	muscimol	gabazine	TTX	muscimol	gabazine
A-1	1	6	46	17 (5 μM)	5 (0.2 mM)	6 (0.1 μM)	18,662	1,270	356	789	2.61 ± 1.89	5.49 ± 5.84^a^	2.76 ± 0.56	2.46 ± 0.54	3 (increase)	1 (increase)	3 (increase)
A-2	2	5	30	8 (2.5 μM)	2 (0. 2 mM)	3 (0.1 μM)	21,279	1,294	121	666	5.34 ± 1.26	6.94 ± 1.54^a^	5.48 ± 1.84	5.10 ± 1.64^a^	1 (decrease)	0	0
A-3	2	7	33	11 (5 μM)	2 (0.2 mM)	13 (0.1 μM)	29,919	4,943	1,405	633	2.89 ± 0.73	3.26 ± 1.05^a^	2.86 ± 0.75	3.28 ± 0.76^a^	1 (increase)	0	0
A-4	1	5	18	8 (5 μM)	2 (1.0 mM)	9 (0.2 μM)	51,778	2,186	2,082	12,055	4.00 ± 1.06	5.57 ± 1.91^a^	4.85 ± 2.01^a^	4.11 ± 1.15^a^	0	0	0
A-5	4	3	19	7 (5 μM)	3 (1.0 mM)	2 (0.1 μM)	7,441	1,576	296	420	19.94 ± 9.60	22.25 ± 12.41^a^	23.04 ± 11.97^a^	20.88 ± 8.77^a^	0	0	0

LMAN, lateral magnocellular nucleus of the anterior neostriatum; SD, standard deviation; TTX, tetrodotoxin; PBS, phosphate-buffered saline.

^a^P < 0.05, Kruskal–Wallis ANOVA test and Tukey HSD post-hoc test, compared to control (PBS) infusion.

**Table 2:** Demographics of the Bengalese finches with microdialysis probe implantations in bilateral Area X.

Bird ID	Number of repeated syllable types	Number of branch points	Days recorded under infusion				Number of song bouts recorded under infusion				Number of syllables in repetitions under infusion Average ± SD				Number of branch points where transition entropy changes significantly^a^		
			PBS	TTX	muscimol	gabazine	PBS	TTX	muscimol	gabazine	PBS	TTX	muscimol	gabazine	TTX	muscimol	gabazine
B-1	1	10	18	6 (5 μM)	2 (1.0 mM)	3 (0.2 μM)	8,001	447	333	1,712	5.13 ± 1.51	6.75 ± 3.24^a^	5.79 ± 1.81^a^	4.95 ± 1.29^a^	0	0	0
B-2	3	10	19	7 (5 μM)	3 (1.0 mM)	3 (0.2 μM)	5,808	608	285	911	5.68 ± 2.38	6.85 ± 2.95^a^	6.03 ± 2.97^a^	5.79 ± 2.40	0	0	1 (increase)
B-3	2	5	45	14 (5 μM)	8 (0.2 mM)	7 (0.1 μM)	52,545	7,375	8,000	6,964	5.01 ± 2.19	5.49 ± 2.71^a^	5.08 ± 2.20^a^	5.06 ± 2.43	1 (increase)	0	0
B-4	2	6	46	14 (5 μM)	8 (0.2 mM)	10 (0.1 μM)	27,704	3,402	3,582	4,682	4.27 ± 1.93	4.45 ± 1.92^a^	4.13 ± 1.80	4.19 ± 1.87^a^	2 (decrease)	0	0
B-5	1	5	47	14 (5 μM)	8 (0.2 mM)	10 (0.1 μM)	37,935	3,936	4,116	3,740	4.62 ± 1.84	5.32 ± 2.50^a^	5.13 ± 1.92^a^	4.41 ± 1.82^a^	1 (increase)	0	2 (increase)
B-6	2	4	45	10 (5 μM)	6 (0.2 mM)	10 (0.1 μM)	13,256	3,110	1,140	1,179	4.88 ± 1.54	5.03 ± 1.31^a^	5.34 ± 1.40^a^	4.98 ± 1.62	0	0	1 (increase)
B-7	3	7	35	6 (5 μM)	7 (0.2 mM)	9 (0.1 μM)	25,336	814	1,285	6,081	3.53 ± 0.90	3.61 ± 0.86^a^	4.01 ± 1.37^a^	3.46 ± 0.85^a^	0	1 (decrease)	0
B-8	2	4	46	11 (5 μM)	7 (0.2 mM)	9 (0.1 μM)	47,083	5,306	5,768	9,007	6.50 ± 2.31	7.32 ± 2.34^a^	7.47 ± 2.73^a^	7.01 ± 2.36^a^	0	0	0
B-9	1	6	45	9 (5 μM)	4 (0.2 mM)	9 (0.1 μM)	16,496	370	896	3,490	3.84 ± 1.57	4.52 ± 1.47^a^	4.59 ± 1.71^a^	4.04 ± 1.72^a^	0	0	1 (decrease)

SD, standard deviation; TTX, tetrodotoxin; PBS, phosphate-buffered saline.

^a^P < 0.05, Kruskal–Wallis ANOVA test and Tukey HSD post-hoc test, compared to control (PBS) infusion.

All the analyses, data processing, and statistical analyses were performed using MATLAB. Statistical significance was set at *P* < 0.05.

### Histological verification of the microdialysis probe position

After recording the auditory behavior, the location of the probes for all the Bengalese finches were histologically confirmed. The bilateral probes were infused with ethidium bromide (10 mg/mL, Nacalai Tesque, Japan), and the birds were deeply anesthetized with 300 μL of 20% urethane and perfused transcardially, first with saline and then with a 4% paraformaldehyde solution. The fixed brain blocks were immersed in 30% sucrose for cryoprotection and cut into 40-μm coronal sections using a cryostat (CM1900; Leica Microsystems, Wetzlar, Germany). The correct placement of the microdialysis probe was verified using histological brain sections. Images were acquired using a digital microscope (BZX700; KEYENCE, Osaka, Japan) with a dry objective lens (×10, NA 0.25).

## Results

### Bilateral placement of microdialysis probes in LMAN and Area X of Bengalese finches

Microdialysis probes were bilaterally implanted into the LMAN of five adult Bengalese finches and into Area X of nine Bengalese finches. Before the implantation, electrophysiological recordings were performed to confirm the auditory response to the individual bird’s own song (BOS) in each location. After all experimental sessions, ethidium bromide was infused through the probes before perfusion, and the locations of the probes were histologically identified, confirming that all probes were placed within the target nuclei (Supplementary Figs. 2 and 3).

### Experimental manipulation of LMAN activity by TTX and GABA receptor agonists and antagonists influence syllable repetition in Bengalese finches

**Fig. 1:**
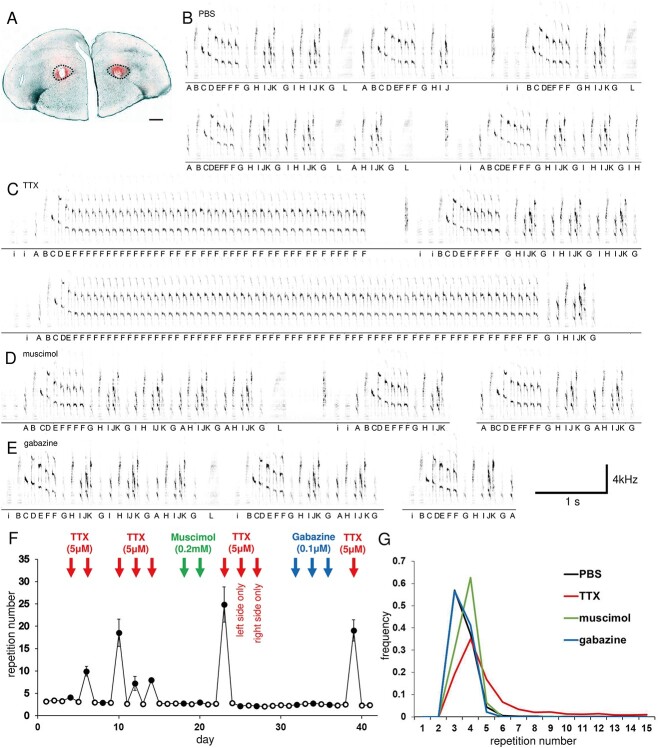
Representative Bengalese finch with microdialysis probe implantations in the bilateral LMAN. A) Location of the bilateral probes in the LMAN (dotted lines) in the brain coronal section. The drug-infused areas were stained by ethidium bromide (red). Scale bar: 500 μm. B) Representative normal control sequences under PBS sham infusion. C) Representative sequences under TTX infusion that include the prominent prolonged syllable repetition. D) Representative sequences under GABA_A_ receptor agonist muscimol infusion that include relatively prolonged syllable repetition. E) Representative sequences under GABA_A_ receptor antagonist gabazine infusion that include relatively reduced syllable repetition. F) Day-by-day chronological consecutive changes in the number of syllable repetitions. Red arrow: TTX infusion days, green arrow: Muscimol infusion days, blue arrow: gabazine infusion days. Error bars represent ± standard error. G) Distributions of the number of syllable repetitions under PBS, TTX, muscimol, and gabazine infusions averaged throughout the entire experimental day. All the figures represent the same Bengalese finch (bird ID, A-1). LMAN, lateral magnocellular nucleus of the anterior neostriatum; TTX, tetrodotoxin; GABA_A_, gamma aminobutyric acid subtype A; PBS, phosphate-buffered saline.

The results of the infusion of PBS (control), TTX, muscimol, or gabazine into the basal ganglia LMAN in five adult Bengalese finches are shown in Fig. 1A–E, and the analysis of the alterations to the syllable sequences (syllable repetitions in particular) is shown in Fig. 1F and G.

In all five Bengalese finches, syllable repetition was significantly prolonged by bilateral TTX infusion (*P* < 0.05, Kruskal–Wallis ANOVA test and Tukey HSD post-hoc test, compared to PBS infusion, Table 1). In two of the five Bengalese finches, syllable repetition was significantly prolonged by bilateral muscimol infusion; however, it did not change in the other three finches. The bilateral gabazine infusion induced mixed effects: syllable repetition significantly shortened in one of the Bengalese finches, prolonged in three finches, and did not change in one finch.

**Fig. 2:**
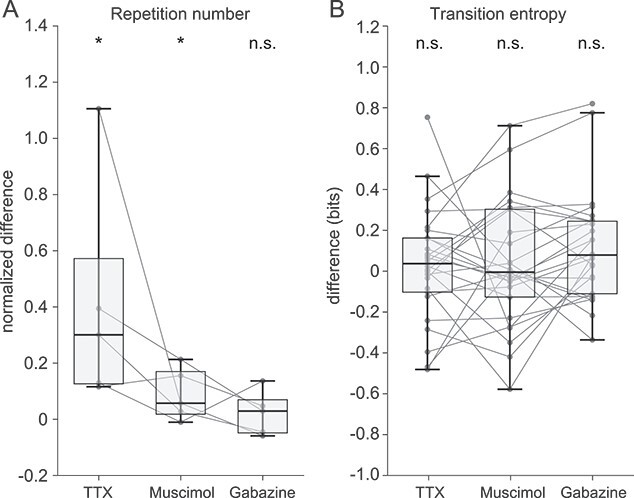
A) Changes in the number of syllable repetitions under TTX, muscimol, and gabazine infusions to the LMAN of nine Bengalese finches. The repetition numbers were averaged throughout the entire experimental day and compared to those under the PBS infusion. The syllable repetition numbers in the sequences were analyzed for the syllable with the longest repeat under PBS infusion in each finch. The normalized differences were calculated as follows: (values under drug infusion − values under PBS infusion)/values under PBS infusion. B) Changes in the transition entropy of the syllable syntax under TTX, muscimol, and gabazine infusions to LMAN. Transition entropy was averaged throughout the entire experimental day and compared to those under the PBS infusion. LMAN, lateral magnocellular nucleus of the anterior neostriatum; TTX, tetrodotoxin; PBS, phosphate-buffered saline. ^*^P < 0.05, Kruskal–Wallis ANOVA test and Tukey HSD post-hoc test, compared to the control (PBS) infusion.

For the five Bengalese finches, bilateral TTX and muscimol infusion significantly prolonged the repetition number (*P* < 0.05, Kruskal–Wallis ANOVA test and Tukey HSD post-hoc test) compared to the control (PBS) infusion; however, bilateral gabazine infusion did not significantly alter the repetition number (Fig. 2A).

To examine the effect of drugs on syllable transition probabilities, changes in transition entropy were examined at each branch point (Supplementary Fig. 4). Among the five Bengalese finches, 26 branch points were identified; the ratios of branch points with significant changes were 19% (increased in 4 of 26 branch points, decreased in 1 of 26), 4% (increased in 1 of 26), and 11% (increased in 3 of 26) with TTX, muscimol, and gabazine infusions, respectively (Supplementary Fig. 5; *P* < 0.05, Kruskal–Wallis ANOVA test and Tukey HSD post-hoc test, compared to PBS infusion, Table 1). Although the effects of TTX, muscimol, and gabazine were observed in a subset of branch points, their effects are weak, and statistical analysis for all 26 branch points did not result in significant changes in the transition entropy (Fig. 2B; *P* > 0.05, Kruskal–Wallis ANOVA test).

The effect of drugs on the acoustic features of syllables was examined in each syllable (Supplementary Fig. 6A). TTX infusion increased the duration in 15% of syllables (5 out of 34), decreased the amplitude in 24% of syllables (8 of 34), and decreased the CV of FF in 25% of syllables (4 of 16) compared to the control (PBS) infusion (Supplementary Fig. 6B; *P* < 0.05, Kruskal–Wallis ANOVA test and Tukey HSD post-hoc test). The effect of muscimol and gabazine infusions on the acoustic features was insignificant in most syllables (see Supplementary Fig. 6B for details).

### Experimental manipulation of Area X activity by TTX and GABA receptor agonists and antagonists influences syllable repetition in Bengalese finches

**Fig. 3:**
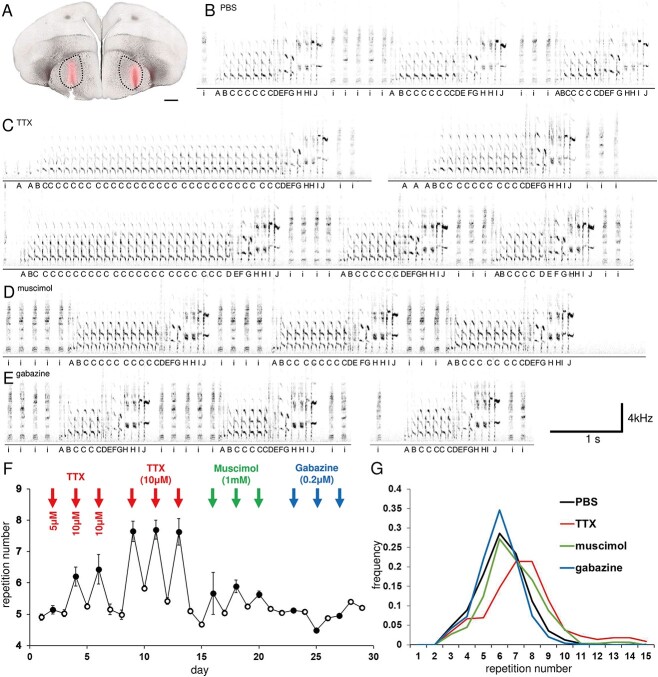
Representative Bengalese finch with microdialysis probe implantations in bilateral Area X. A) Location of the bilateral probes in area X (dotted lines) in the coronal section of the brain. The drug-infused areas were stained by ethidium bromide (red). Scale bar: 500 μm. B) Representative normal control sequences under PBS sham infusion. C) Representative sequences under TTX infusion that include the prominent prolonged syllable repetition. D) Representative sequences under GABA_A_ receptor agonist muscimol infusion that include the relatively prolonged syllable repetition. E) Representative sequences under GABA_A_ receptor antagonist gabazine infusion that include the relatively reduced syllable repetition. F) Day-by-day chronological consecutive changes in the number of syllable repetitions. Red arrow: TTX infusion days, green arrow: Muscimol infusion days, blue arrow: gabazine infusion days. Error bars represent ± standard error. G) Distributions of the number of syllable repetitions under PBS, TTX, muscimol, and gabazine infusions that averaged throughout the entire experimental day. All of the figures are of the identical Bengalese finch (bird ID, B-1). TTX, tetrodotoxin; GABA_A_, gamma aminobutyric acid subtype A; PBS, phosphate-buffered saline.

The results of the infusion of PBS (control), TTX, muscimol, or gabazine in Area X of the basal ganglia (located upstream of LMAN and receiving direct input from HVC) in nine adult Bengalese finches are shown in Fig. 3A–E and Supplementary Fig. 3. The results of the analysis of the alterations to the syllable sequences (syllable repetitions in particular) are shown in Fig. 3F and G.

In all nine Bengalese finches, syllable repetition was significantly prolonged by bilateral TTX infusion (*P* < 0.05, Kruskal–Wallis ANOVA test and Tukey HSD post-hoc test, compared to PBS infusion, Table 2). In eight out of the nine Bengalese finches, syllable repetition was significantly prolonged by bilateral muscimol infusion; however, it did not change in one finch. The bilateral gabazine infusion induced mixed effects; syllable repetition shortened significantly in four of the nine Bengalese finches, prolonged in two finches, and did not change in the other three finches.

**Fig. 4:**
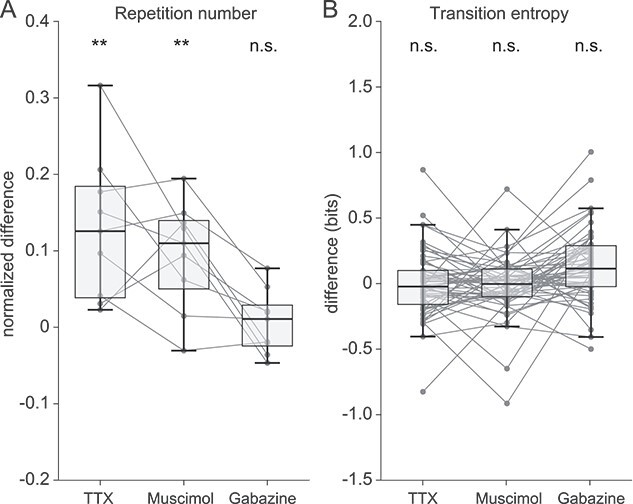
A) Changes in the number of syllable repetitions under TTX, muscimol, gabazine infusion to the Area X of five Bengalese finches. The repetition numbers were averaged throughout the entire experimental day and compared to those under PBS infusion. The syllable repetition numbers in the sequences were analyzed for the syllable with the longest repeat under PBS infusion in each finch. B) Changes in the transition entropy of the syllable syntax under TTX, muscimol, and gabazine infusions to Area X. Transition entropy was averaged throughout the entire experimental day and compared to those under the PBS infusion. ^*^^*^P < 0.01, Kruskal–Wallis ANOVA test and Tukey HSD post-hoc test, compared to control (PBS) infusion.

For the nine Bengalese finches, bilateral TTX and muscimol infusions significantly prolonged the repetition number compared to the control (PBS) infusion (*P* < 0.01, Kruskal–Wallis ANOVA test and Tukey HSD post-hoc test); however, bilateral gabazine infusion did not alter the repetition number significantly (Fig. 4A). These changes in repetition number were more evident for syllables with high repetition numbers than for those with low repetition numbers (Supplementary Fig. 7).

To analyze the effect of drugs on transition entropy, 58 branch points of the nine Bengalese finches were identified; the ratios of branch points with significant changes were 7% (increased in 2 of 58 branch points, decreased in 2 of 58), 2% (decreased in 1 of 58), and 9% (increased in 4 of 58, decreased in 1 of 58) with TTX, muscimol, and gabazine infusions, respectively (*P* < 0.05, Kruskal–Wallis ANOVA test and Tukey HSD post-hoc test, compared to PBS infusion, Table 2). Although the effects of TTX, muscimol, and gabazine were observed in a subset of branch points, their effects are weak, and statistical analysis for all 58 branch points did not result in significant changes in the transition entropy (Fig. 4B; *P* > 0.05, Kruskal–Wallis ANOVA test).

The effect of drugs on the acoustic features of syllables was examined in each syllable (Supplementary Fig. 6C). TTX infusion to Area X increased the duration in 17% of syllables (13 of 78), increased the amplitude in 9% of syllables (7 of 78), and decreased the CV of FF in 13% of syllables (5 of 39) compared to the control (PBS) infusion (Supplementary Fig. 6D; *P* < 0.05, Kruskal–Wallis ANOVA test and Tukey HSD post-hoc test). In a subset of syllables, TTX infusion also affected the entropy (19%; 11 increased and 4 decreased out of 78) and mean frequency (12%; 6 increased and 3 decreased out of 78); however, the effect varied among syllables. The effect of muscimol and gabazine infusions on the acoustic features was insignificant in most syllables (see Supplementary Fig. 6D for details).

## Discussion

In this study, we pharmacologically manipulated the basal ganglia of Bengalese finches. Inhibition of Area X and LMAN by TTX and muscimol resulted in the prolongation of repetitive sequences, while the transition entropy was affected only at a subset of branch points. Our results suggest that the syntactic structure is determined not only by higher motor regions but also by the active involvement of the basal ganglia.

The basal ganglia are believed to play a role in habitual repetitive movements. In rodents, striatal neurons encode the initiation and termination of repetitive behaviors acquired by training (Jin and Costa 2010; Martiros et al. 2018). In addition, abnormal function taking place in basal ganglia disorders induce repetitive movements. Basal ganglia impairment results in motor or speech disorders, including Parkinson’s disease in humans (Bocanegra et al. 2015). Tics, a major symptom of Tourette syndrome, are characterized by rapid, repetitive involuntary movements; and they can be induced by pharmacological manipulation of the GABAergic tone in the striatum in rodents (Gittis et al. 2011; Bronfeld et al. 2013) and monkeys (Bronfeld et al. 2011). These previous studies indicate that the basal ganglia are responsible for executing repetitive movements, and that GABAergic intervention may induce unintended repetition. In mice, a subregion in the dorsolateral striatum is responsible for grooming sequences (Cromwell and Berridge 1996). Our histological examination showed the probes had been randomly placed within Area X (Supplementary Fig. 3), but whether Area X includes a subregion responsible for song syntax remains unclear.

Songbirds have been used as model animals to study the role of the cortico-basal ganglia circuits on sequential movements. Juvenile zebra finches produce immature birdsongs with high variability between individual renditions, which decreases after bilateral LMAN lesioning (Scharff and Nottebohm 1991). In contrast, bilateral infusion of bicuculline to LMAN induces sequence variability in stereotypic songs of adult zebra finches (Hamaguchi and Mooney 2012). Adult Bengalese finches possess variable syntax in syllable sequence vocalization, including syllable repetition, with a capacity to modulate the sequencing to avoid aversive stimuli (Warrant et al. 2012).

Several lines of evidence support the idea that the basal ganglia control syllable sequence in Bengalese finches. HVC of Bengalese finches, an analog of the supplementary motor cortex, encodes syntactic information (including syllable repetition) and conveys this information onto Area X of the basal ganglia (Fujimoto et al. 2011). The output of the basal ganglia influences songs in a sequence-dependent manner (Tian and Brainard 2017). Transient prolongation of repetitive sequences in Bengalese finches was induced by bilateral lesions in Area X (Kobayashi et al. 2001). Our results are consistent with the idea that the basal ganglia modulate syllable sequencing, and confirm previous results reversibly by using microdialysis probes.

However, lesion studies to examine the influence of the cortico-basal ganglia circuits on syllable sequencing have yielded contradictory results (Hampton et al. 2009). The syllable repetition prolongation effect of bilateral lesions in Area X (Kobayashi et al. 2001) was transient. In a previous study (Hamaguchi and Mooney 2012) wherein a reversible technique was used, inactivating LMAN increased sequence variability and transition entropy in adult zebra finches. Taking together these results and considering that our study and that by Hamaguchi and Mooney (2012) used reversible techniques, the effect of syllable repetition prolongation by suppression of the basal ganglia might be observed on a short time scale. A lesion study (Hampton et al. 2009) examined the fixed and stable states after the lesion, resulting in no changes in song syntax.

The current technique is reversible; thus, the song should recover to its normal state. However, in our study, drug flush-out by PBS was performed after each recording, resulting in a subtle decay of the drug effect on vocalization in the experimental period (Supplementary Fig. 1).

A previous study (Wittenbach et al. 2015) demonstrated that deafening reduces syllable repetitions. The auditory feedback is processed by both Area X and LMAN (Brainard and Doupe 2000); thus, deafening presumably affects and perturbs the activities of these two regions. This may change syllable repetition vocalization.

Another previous study on syllable repetitions in Bengalese finches (Zhang et al. 2017) demonstrated that HVC cooling reduces syllable repetition. This result implies that syllable repetition is partially controlled by the premotor nucleus, HVC. In conjunction with our results, it is probable that HVC and the basal ganglia affect or modulate each other (bidirectionally) or at least unidirectionally during the syllable repetition vocalization.

A transgenic zebra finch in which a huntingtin mutant is dominantly expressed in the song system (including Area X and LMAN) has been reported to exhibit syntax stereotypy, simplification, and prolongation of syllable repetition (Liu et al. 2015). A recent study that utilized selective neuron ablation by injection of viral particles into areas related to the song system revealed that ablation of HVC_X_ neurons induces syntax deterioration in zebra finches (Sánchez-Valpuesta et al. 2019). Considered together with a previous study indicating that HVC_X_ neurons in the Bengalese finch encode syntactic information (including syllable repetition) (Fujimoto et al. 2011), our results in the Bengalese finches suggest that Area X and LMAN in the basal ganglia are involved in repetitive syntax regulation in songbirds.

In this study, the effect of gabazine was unclear, although gabazine effects were partially positively suspected particularly in Area X (i.e. bird ID B-1, B-4, B-5, and B-7). The high neural toxicity of gabazine prevents high-dose infusion, and this limitation could explain the lack of a significant effect of this compound observed in Bengalese finches.

Although the position of the microdialysis probes was electrophysiologically and histologically verified in this study, different molecular weights or tissue affinity of the drugs could alter the affected volume within the targeted brain region. Most importantly, this study cannot exclude the possibility that drugs may have unintentionally spread to neighboring brain regions. This limitation could be overcome in future studies using transgenic animals and cell type-specific genetic manipulations, such as virus-mediated neuronal ablation. The genetic approaches currently available for use in songbirds are relatively limited, but progress has been made (Abe et al. 2015; Tanaka et al. 2016). Studies involving genetic manipulation as part of their design and/or reversible manipulations, such as in the present study, are warranted in the future.

## Conclusion

Inhibition of Area X and LMAN in the basal ganglia of songbirds using tetrodotoxin or GABAergic manipulation, including inhibition enhancement through muscimol, affects repetitive vocalization in Bengalese finches, suggesting that the basal ganglia are involved in the regulation of repetitive syllables during syntactic vocalization.

## Author contributions

Hisataka Fujimoto (Conceptualization, Data curation, Formal analysis, Funding acquisition, Investigation, Methodology, Project administration, Resources, Software, Supervision, Validation, Visualization, Writing—original draft), and Taku Hasegawa (Formal analysis, Investigation, Methodology, Software, Supervision, Validation, Visualization, Writing—review & editing)

## Funding

This work was supported in part by the Japan Society for the Promotion of Science (JSPS) KAKENHI (grant numbers 25861630 and 16K18378 to HF). The data supporting the findings of this study are available from the corresponding author, HF, upon reasonable request.

*Conflict of interest statement.* None declared.

## Supplementary Material

SupportingInformation_tgad016
