# Supplementary material for: Reversible inhibition of the basal ganglia prolongs repetitive vocalization but only weakly affects sequencing at branch points in songbirds
Source: Cereb Cortex Commun. 2023 Aug 17;4(3):tgad016. doi: 10.1093/texcom/tgad016 (PMC10477706; doi:10.1093/texcom/tgad016)
Supplement: SupportingInformation_tgad016 [file supportinginformation_tgad016.pdf]

Supporting information

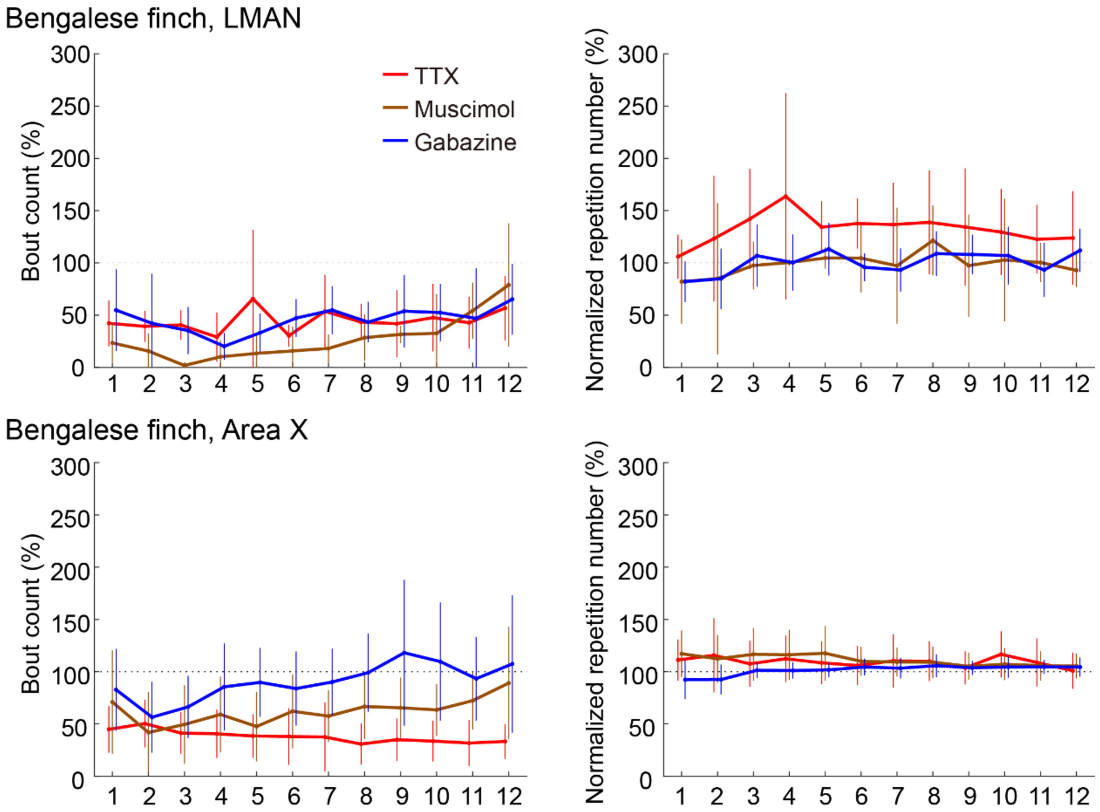

Supplementary Figure 1. Diurnal variation in song production and syllable repetition in response to drug infusions. Bout counts and repetition numbers under TTX, muscimol, and gabazine infusions have been normalized to those observed under PBS infusion. The repetition number was calculated for the syllable of the longest repeat in each bird. Error bars represent mean ± standard deviation. TTX, tetrodotoxin; PBS, phosphate buffered saline.

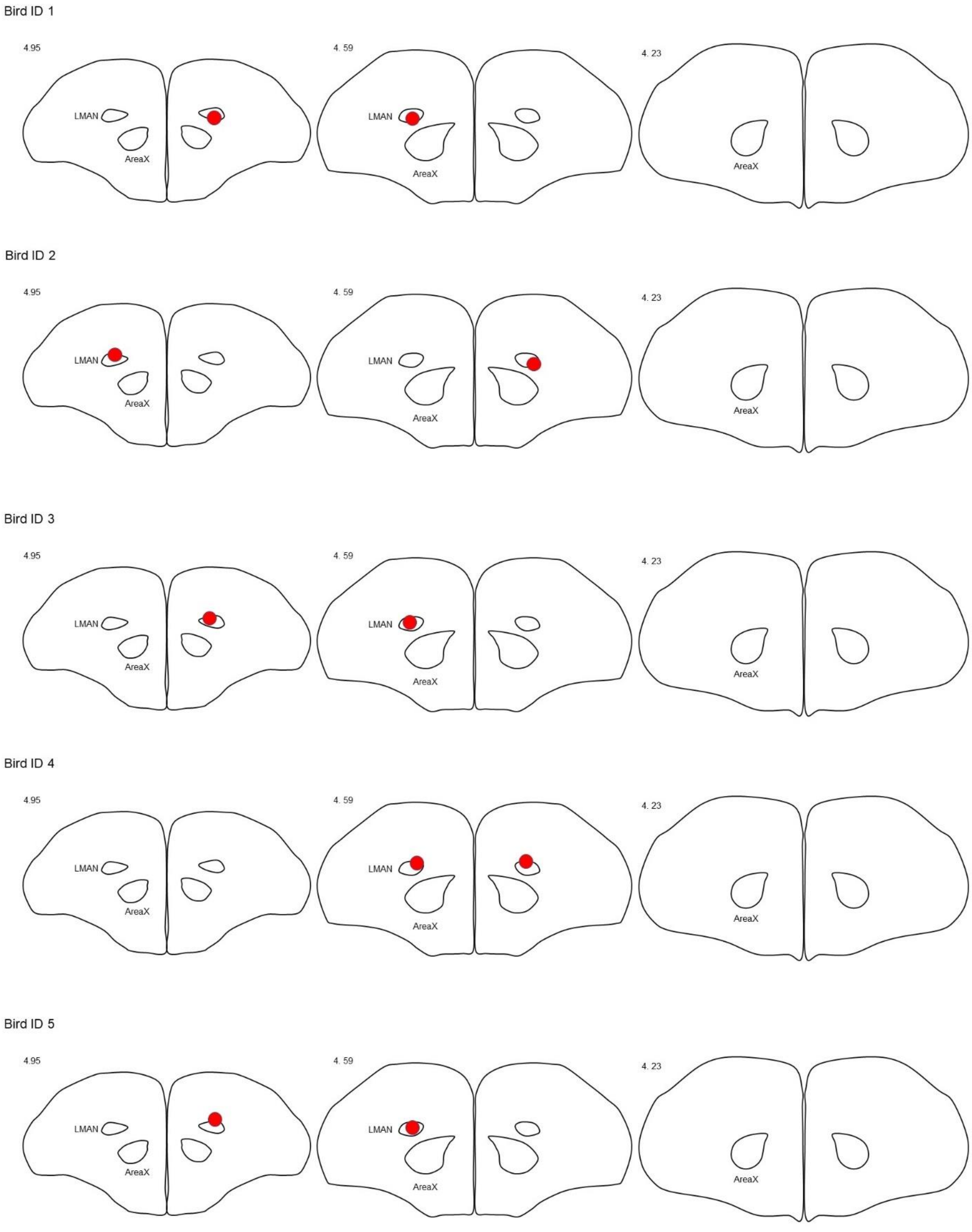

Supplementary Figure 2. Locations of the probe center (red circles) in the LMAN of all five Bengalese finches tested in this study. The probe locations were visualized by infusing

ethidium bromide before perfusion. Red circles indicate the gravity centers of the ethidium bromide staining on three different coronal sections at 4.95, 4.59, and 4.23 mm rostral to the caudal edge of the bifurcation of the midsagittal sinus. LMAN, lateral magnocellular nucleus of the anterior neostriatum.

Bird ID 6

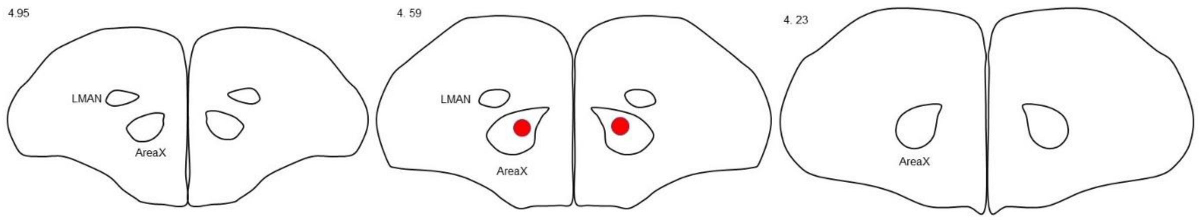

Bird ID 7

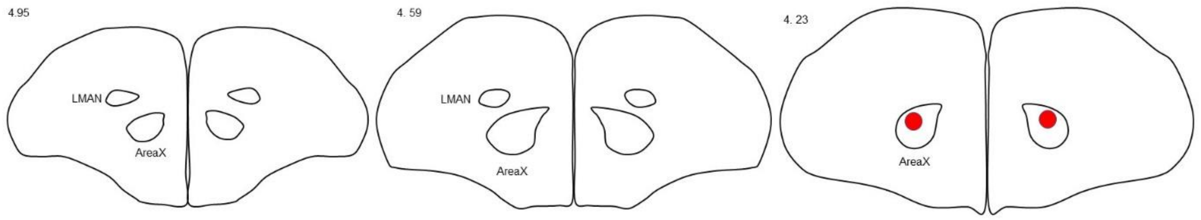

Bird ID 8

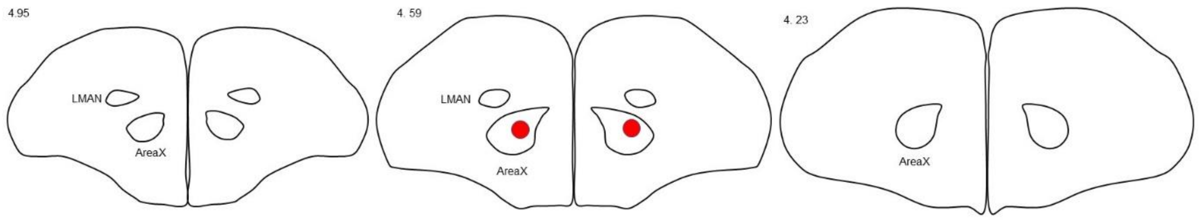

Bird ID 9

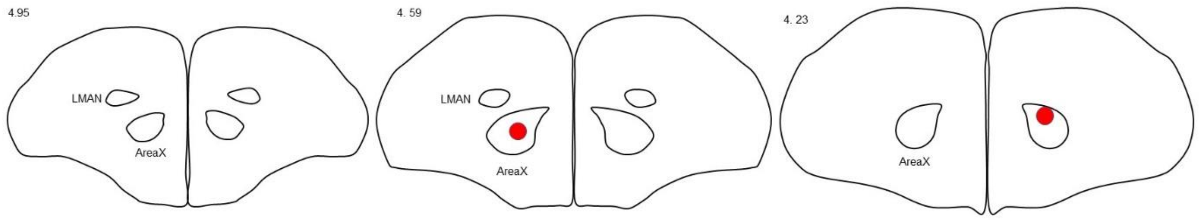

Bird ID 10

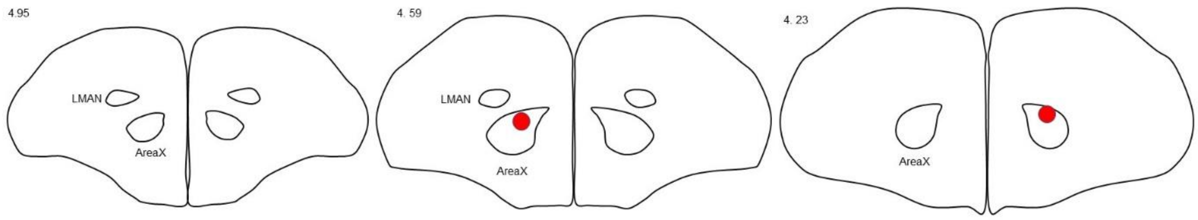

Bird ID 11

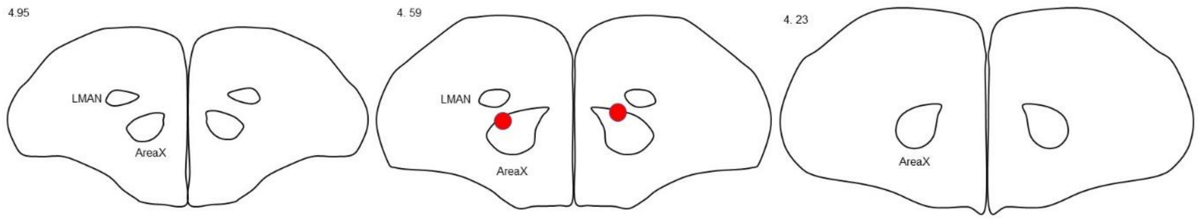

Bird ID 12

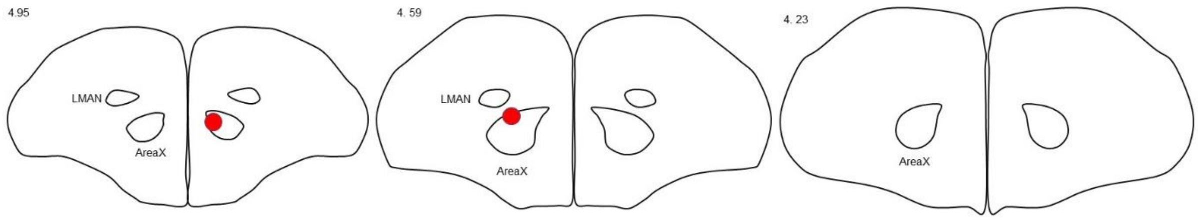

Bird ID 13

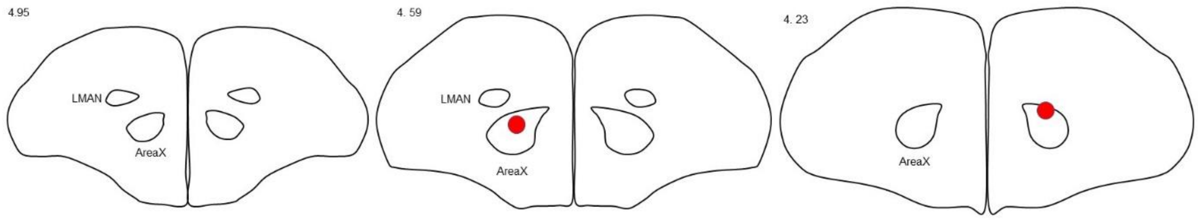

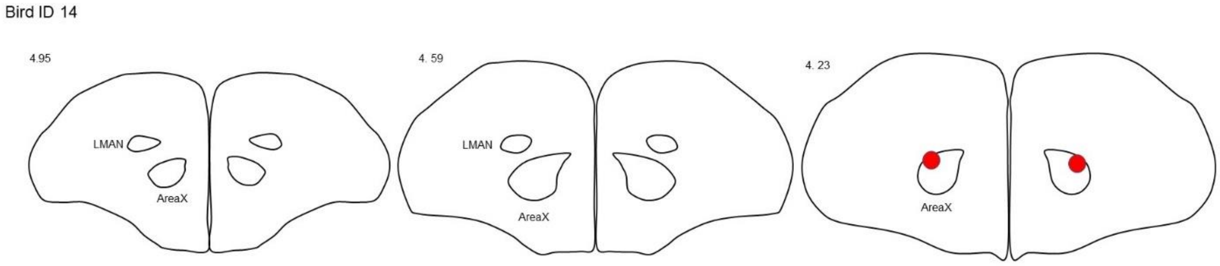

Supplementary Figure 3. Locations of the probe center (red circle) in Area X of all nine Bengalese finches tested in this study. The probe locations were visualized by infusing ethidium bromide before perfusion. Red circles indicate the gravity centers of the ethidium bromide staining on three different coronal sections at 4.95, 4.59, and 4.23 mm rostral to the caudal edge of the bifurcation of the midsagittal sinus.

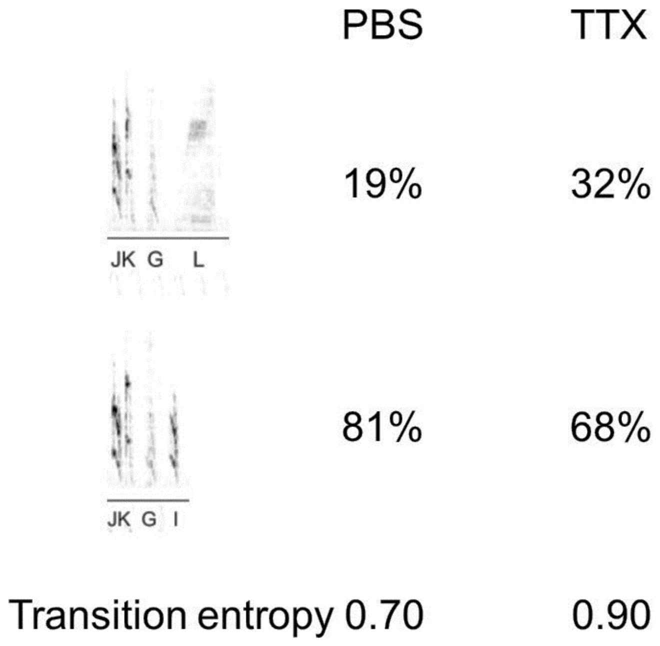

Supplementary Figure 4. Example of a branch point. The panels on the left show spectrograms of the transitions following the branch point sequence (“JKG”). The transition probabilities (%) for each of the branch point transitions following PBS or TTX infusions are shown on the right side of the figure. The change in transition entropy is observed according to the transition probability. TTX, tetrodotoxin; PBS, phosphate buffered saline.

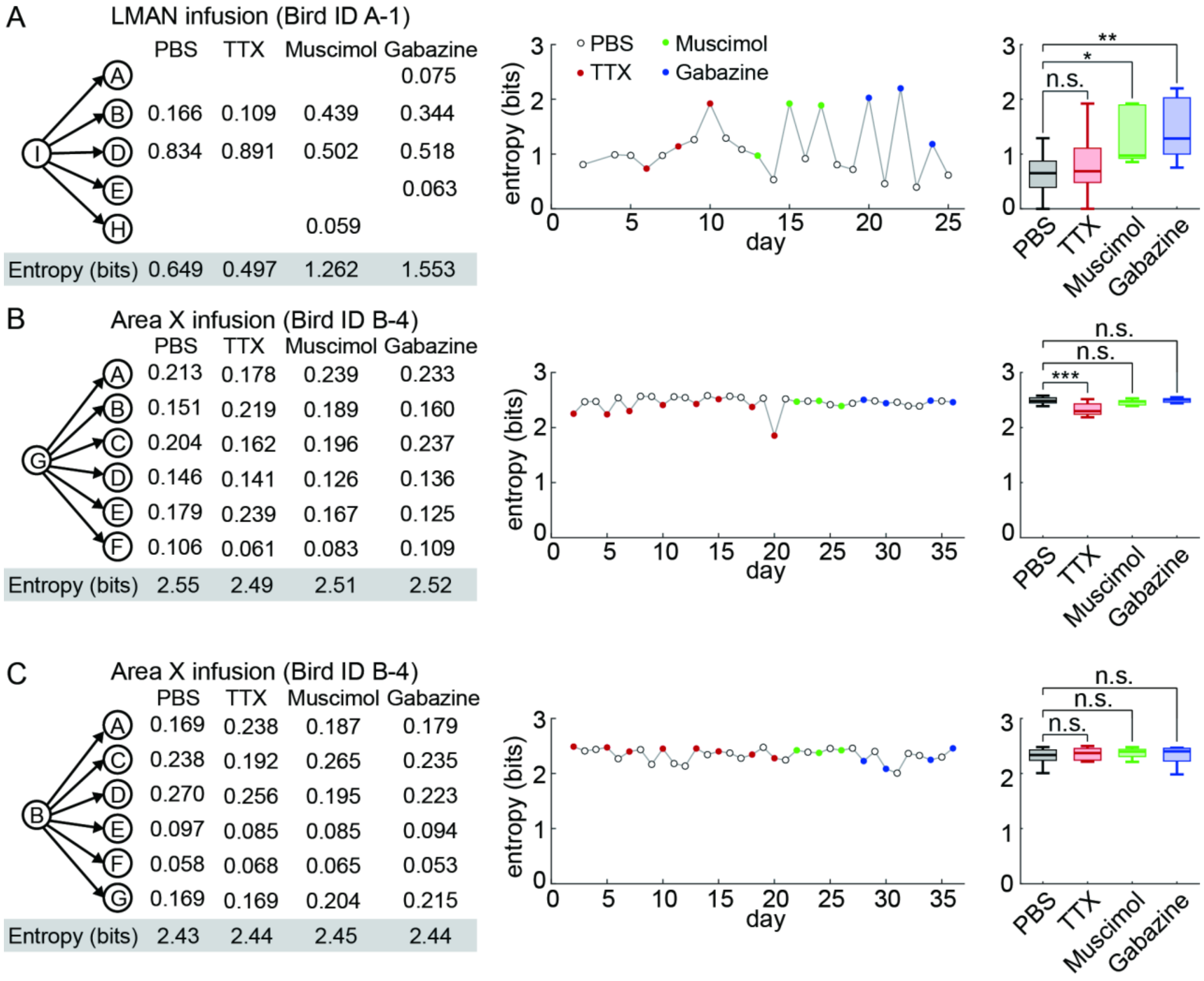

Supplementary Figure 5. Examples of changes in transition probabilities and entropy in response to TTX, muscimol, and gabazine infusions. **A**) Graphical representation of a branch point of a Bengalese finch with drug infusions into bilateral LMAN (left). Numbers indicate transition probabilities and entropy calculated from all daily sessions. Chronological changes in transition entropy with drug infusions (middle) and a bar plot representing distributions of transition entropy across sessions (right). **B-C**) Same as **A** but for a branch point of another Bengalese finch with drug infusions into bilateral Area X. n.s. (insignificant), $P \geq 0.05$, $*P < 0.05$, $**P < 0.01$, $***P < 0.001$, Kruskal–Wallis ANOVA test with Tukey HSD post-hoc test compared to the PBS infusion.

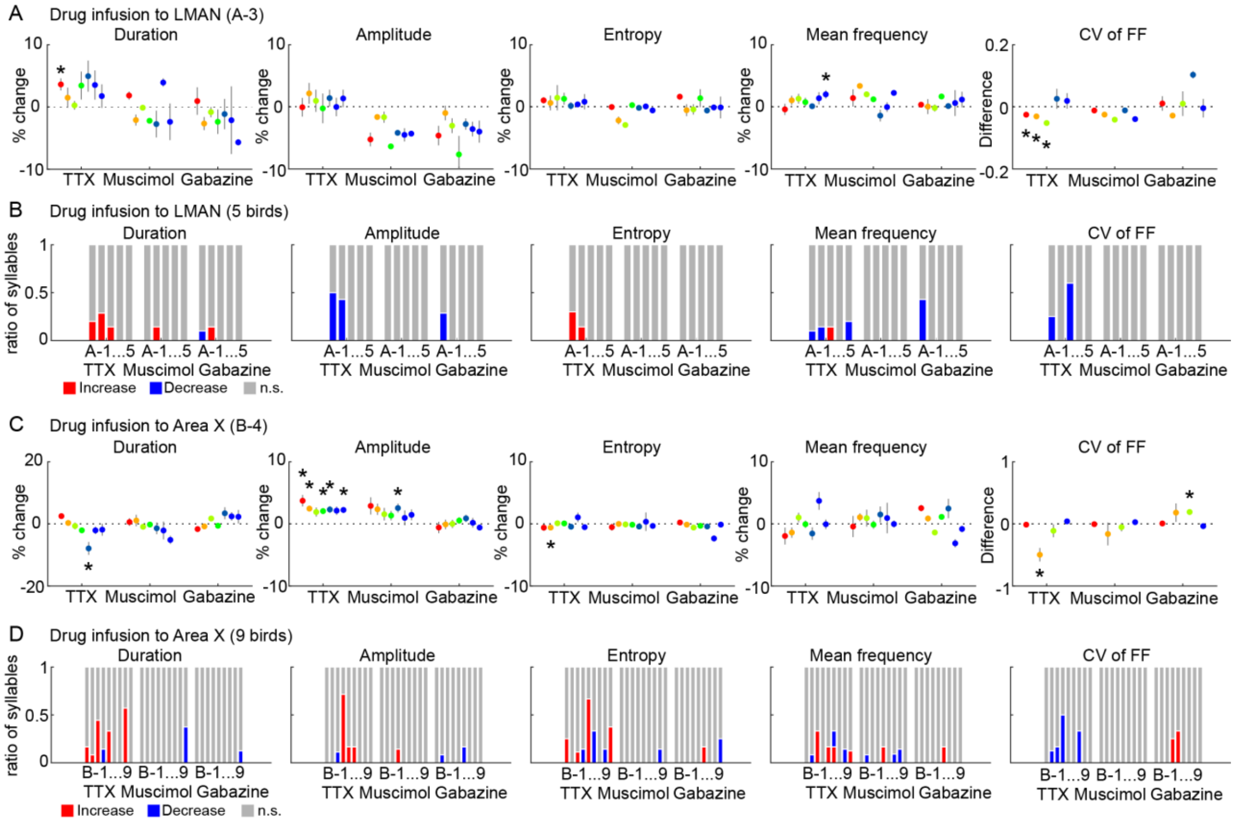

Supplementary Figure 6. Effects of drug infusions on acoustic features. Mean values of duration, amplitude, entropy, mean frequency, and coefficient of variation of fundamental frequency (CV of FF) in each session following drug infusion were normalized to those observed following PBS infusion. **A**) Example of changes in the acoustic features of a Bengalese finch with microdialysis probe implantation in the bilateral LMAN (Bird ID: A-3 in Table 1). Acoustic features were calculated for each experimental day, and statistical analysis was performed for each syllable. Dots and vertical lines indicate means and standard errors, respectively. The colors of the dots are consistent for the same syllables. Note that the CV of FF was calculated for only a subset of syllables with stable harmonic components (see Materials and Methods). **B**) Ratio of syllables with significant changes in acoustic parameters in response to drug infusions to the LMAN (Bird ID: A-1 to A-5). **C**) Same as **A** but for a Bengalese finch with microdialysis probe implantation in bilateral Area X (Bird ID: B-4 in Table 2). **D**) Same as **B** but for drug infusions to Area X (Bird ID: B-1 to B-9). *$P < 0.05$, Kruskal–Wallis ANOVA test with Tukey HSD post-hoc test compared to values from the PBS infusion.

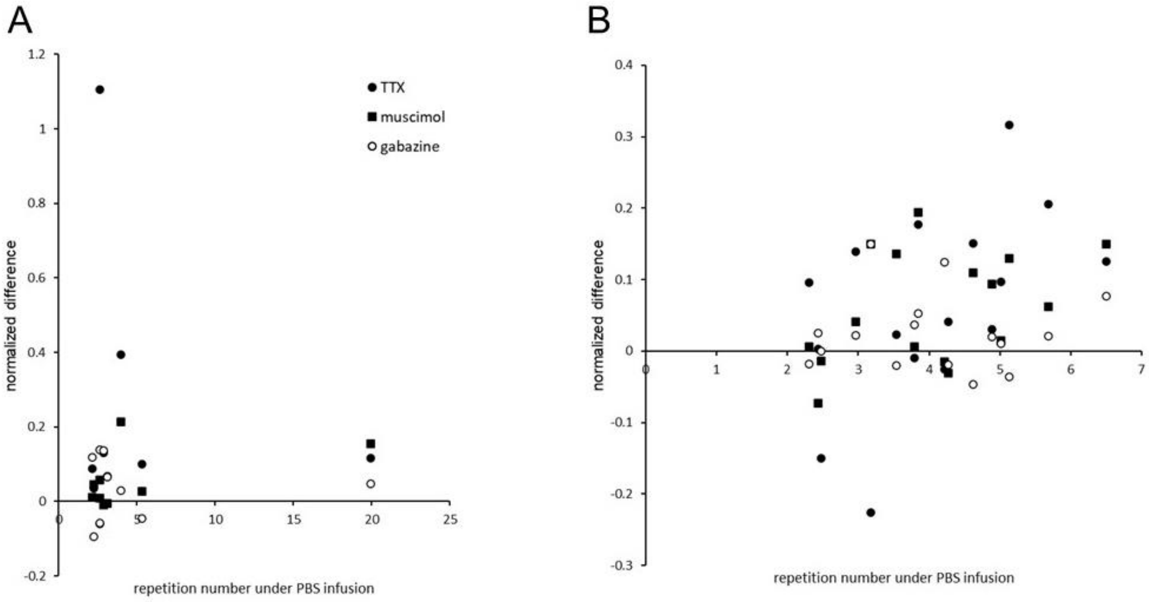

Supplementary Figure 7. Changes in the numbers of syllable repetitions in response to TTX, muscimol, and gabazine infusions in Bengalese finches. The drugs were bilaterally infused in LMAN of five Bengalese finches (A) and in Area X of nine Bengalese finches (B). All of the syllables with more than two repetitions (in average) are presented. Repetition numbers in each bird were compared and normalized to those in control (PBS) sessions.
